# Genetic Characterization of Feline Parvovirus Isolate Fe–P2 in Korean Cat and Serological Evidence on Its Infection in Wild Leopard Cat and Asian Badger

**DOI:** 10.3389/fvets.2021.650866

**Published:** 2021-05-07

**Authors:** Young Ji Kim, Sun-Woo Yoon, Jin Ho Jang, Dae Gwin Jeong, Beom Jun Lee, Hye Kwon Kim

**Affiliations:** ^1^Infectious Disease Research Center, Korea Research Institute of Bioscience and Biotechnology, Daejeon, South Korea; ^2^College of Veterinary Medicine, Chungbuk National University, Chungju, South Korea; ^3^College of Bioscience, University of Science and Technology, Daejeon, South Korea; ^4^Department of Wildlife Disease, College of Veterinary Science, Jeju National University, Jeju, South Korea; ^5^Chungnam Wild Animal Rescue Center, Kongju National University, Yesan, South Korea; ^6^Department of Microbiology, College of Natural Science, Chungbuk National University, Cheongju, South Korea

**Keywords:** feline parvovirus, feline panleukopenia, leopard cat, Asian badger, serum neutralization

## Abstract

Feline parvovirus (FPV) is a small, non-enveloped, single-stranded DNA virus that infects cats. We recently isolated a feline parvovirus Fe–P2 strain from a dead stray cat in Iksan, 2017. Its partial genomic sequence (4,643 bases) was obtained, and phylogenetic analysis based on the VP2 nucleotide sequence showed that the FPV Fe-P2 strain was closely related to the FPV isolate Gigucheon in cat, 2017 (MN400978). In addition, we performed a serum neutralization (SN) test with the FPV isolates in various mammalian sera. These were from raccoon dog, water deer, Eurasian otter, Korean hare, leopard cat, and Asian badger, which were kindly provided by Chungnam Wild Animal Rescue Center. Notably, serological evidence of its infection was found in Asian badger, *Meles leucurus* (2/2) and leopard cat, *Prionailurus bengalensis* (5/8) through SN tests, whereas there was no evidence in raccoon dog, water deer, Eurasian otter, and Korean hare based on the collected sera in this study. These findings might provide partial evidence for the possible circulation of FPV or its related viruses among wild leopard cat and Asian badger in Korea. There should be additional study to confirm this through direct detection of FPVs in the related animal samples.

## Introduction

Feline parvovirus (FPV) is a single-stranded DNA virus which is a variant of *Carnivore protoparvovirus 1*, belonging to the genus *Protoparvovirus* within the family *Parvoviridae*. A range of serious condition (often lethal disease, inducing vomiting, enteritis, diarrhea, and acute lymphopenia) in young animal is closely involved the *Carnivore protoparvovirus 1*.

FPV is the main causative agent for feline panleukopenia, which can also be caused by canine parvovirus (CPV) variants, CPV-2a, 2b, and 2c (1, 2). CPV-2 can only infect dogs, whereas its variant can infect cats (1, 3). Thus, parvovirus members of *Carnivore protoparvovirus 1* might be one of the host range variants (4). Also, mink enteritis virus (MEV) and raccoon parvovirus (RaPV) are included in that.

FPV can infect not only domestic cats, but also other species such as raccoons, foxes, and minks (5). Previous findings have reported the detection of FPV-sequences in tissues of the African wild cat and in feces of both cheetahs and honey badgers (6). In Italy, FPV was detected in red foxes (*Vulpes vulpes*) (2.8%, 7/252) and Eurasian badgers (*Meles meles*) (10%, 1/10), and in Portugal, parvovirus DNA was detected in Egyptian mongoose (57.8%), red fox (78.9%), and stone marten (75%) (7, 8). Although FPV can infect diverse animal species, it is difficult to confirm the FPV-infection cases due to the genetic similarity and cross-reactivity between FPV and CPV (3).

The parvovirus contains two open reading frames. One is the codes for non-structural proteins (NS1 and NS2) and the other is the codes for structural viral proteins (VP1 and VP2). Several amino acid changes in the structural protein VP2, its major capsid protein, were associated with host specificity and antigenic properties for the parvovirus (9–11). Based on VP2 gene analysis, there were three genetic clusters (G1, G2, and G3) of FPV around the world. In Korea, FPVs belonging to both the G1 and G2 clusters were found (12). In Korea, 2% of 200 cats were FPV-positive in Seoul (11). To date there has been no study about the possible interspecies transmission of FPV among wild mammals in Korea.

In this study, a recent feline parvovirus Fe-P2 strain was isolated from a fecal swab from a stray cat carcass found in Iksan, 2017. Using this isolate, its genomic sequence was obtained, and serum neutralization (SN) tests were performed with various sera from several wild mammals rescued by the Chungnam Wild Animal Rescue Center between 2016 and 2018. Through these experiments, this study provide evidence for the possible interspecies transmission of FPV (or its related viruses) among wild carnivores in Korea.

## Materials and Methods

### Virus Isolation

**Figure 1:**
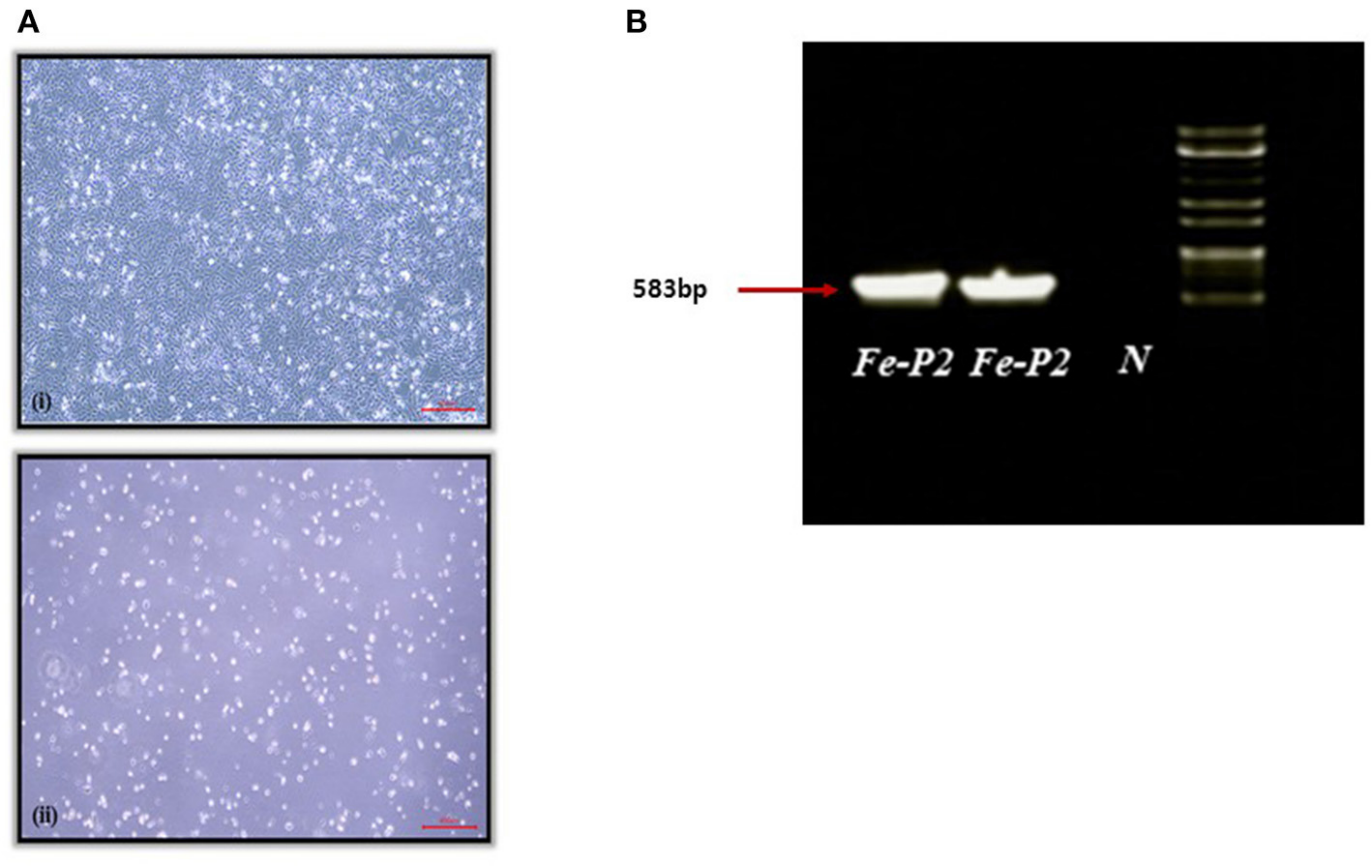
**(A)** Cytopathogenic effect of feline parvovirus (FPV) Fe-P2 on cell culture. (7 days); (i) Uninfected cell control, (ii) CRFK infected with FPV Fe-P2, **(B)** Screening of clinical samples by PCR assay using CPV-2a and b primers. A single DNA amplicon of 583 bp is shown after using the CPV-2a and b primer pair, Fe-P2, FPV isolate; N, negative control.

A fecal swab was collected from a dead stray cat found in Iksan, Korea, 2017. The swab was transported to the lab with virus transport medium (Noble Bio. Co., Ltd., Hwasung, Korea). The transport medium containing the fecal swab was centrifuged at 3,000 g for 20 min at 4°C, and the supernatant was filtered through a 0.22 μm syringe filter, MF-Millipore™ Membrane Filter (Merck, Darmstadt, Germany). The filtered supernatant was inoculated on the monolayer of CRFK cells and incubated for 1 h, followed by phosphate buffered saline (PBS) washing. The inoculated cells were further incubated for 7 d with DMEM, supplemented with 2.5% fetal bovine serum (FBS) and cytopathic effect (CPE) was observed after two blind passages (Figure 1A). After freezing and thawing, the supernatant was aliquoted for further testing.

### Polymerase Chain Reaction and Sequencing

DNA was extracted from the virus stock using QIAamp DNA Mini Kit (QIAGEN, Hilden, Germany). First, PCR targeting VP2 was performed by using primer sets: 555-F† 5′-CAGGAAGATATCCAGAAGGA-3′ and 555-R^*†*^ 5′-GGTGCTAGTTGATATGTAATAAACA-3′ (13).

A PCR 583 bp amplicon (Figure 1B) was obtained and further sequenced by Cosmogenetech, Seoul, Korea. Genomic sequencing was performed using the designed primers based on the reference FPV genomic sequence (MN683826) and PCR-positive sequences. Primer information is presented in Table 1.

**Table 1:** Primers used for genotyping and sequence analysis.

**Primer**	**Sequence (5′ → 3′)**	**Sense**	**Position (Fe–P2)**
555-F^a^	CAGGAAGATATCCAGAAGGA	+	3,841–3,860
555-R^a^	GGTGCTAGTTGATATGTAATAAACA	–	4,423–4,399
FPVin-F4	GGCAATTGCTCCCGTATT	+	2,465–2,482
FPVin-R4	AGCCATGTTTCCTTTAACTGCAG	–	2,915–2,893
FPV-F3	CAGAATCTGCTACTCAGCC	+	3,085–3,103
FPV-R5	ACCAACCACCCACACCAT	–	4,823–4,806
NSF-1	CTGGCAACCAGTATACTG	+	115–132
NSR-1	GCTTGTGCTATGGCTTGAGC	–	1,357–1,338

a *Primers for amplification of the 583 bp products represent a parvovirus-specific VP2 gene (6)*.

### Genetic Analysis

Phylogenetic analysis based on the nucleotide sequences showed that the detected feline parvoviruses belonged to the genera Protoparvovirus. The obtained genomic sequences of the FPV Fe-P2 strain were further analyzed with related sequences from GenBank using BioEdit (14) and MEGA version 7.0 (15) tools. Phylogenetic trees based on the genomic sequences and VP2 were drawn with the maximum likelihood method, with 1,000 replicates of bootstrap sampling and using the Kimura 2-parameter model using MEGA version 7.0. Genomic sequence data generated in this study have been deposited in GenBank under the accession number MN683826.

### Serum Neutralization Test

A total of 109 sera from wild mammals, including raccoon dog, water deer, Eurasian otter, Korean hare, leopard cat, and Asian badger, were obtained from rescued wild animals from the Chungnam Wild Animal Rescue Center. The information on the animal species and collection dates is presented in Table 2.

**Table 2:** Wild animal sera information for the serum neutralization test.

Animal species^*^	**Collected year**	**No. of sera**
Raccoon dog (*Nyctereutes procyonoides koreensis*)	2016–2018	96
Asian badger (*Meles leucurus*)	2016–2017	2
European Otter (*Lutra lutra*)	2018	1
Leopard cat (*Prionailurus bengalensis*)	2018	8
Korean hare (*Lepus coreanus*)	2017	1
Water deer (*Hydropotes inermis*)	2018	1
	Total sera	109

* *A total of six mammalian species: Asian badger (Meles leucurus), leopard cat (Prionailurus bengalensis), water deer (Hydropotes inermis), Eurasian otter (Lutra lutra), raccoon dog (Nyctereutes procyonoides koreensis), and Korean hare (Lepus coreanus)*.

For the SN test, the sera samples were first inactivated at 56°C for 30 min. The inactivated sera were two-fold diluted from an initial dilution of 1:10 with DMEM in 96 well-plates. The diluted sera were then mixed with the same volume of FPV Fe–P2 isolate in 200 Tissue Culture Infective Dose (TCID_50_)/50 μl. The mixture was incubated for an hour at 37°C and 100 μl of that were transferred to the CRFK monolayers with 5% FBS in a 96 well-cell culture plate, followed by incubation at 37°C for 4–5 days. Then, the plates were examined for CPE.

## Results

### Genetic Characterization of the Feline Parvovirus Fe–P2

**Figure 2:**
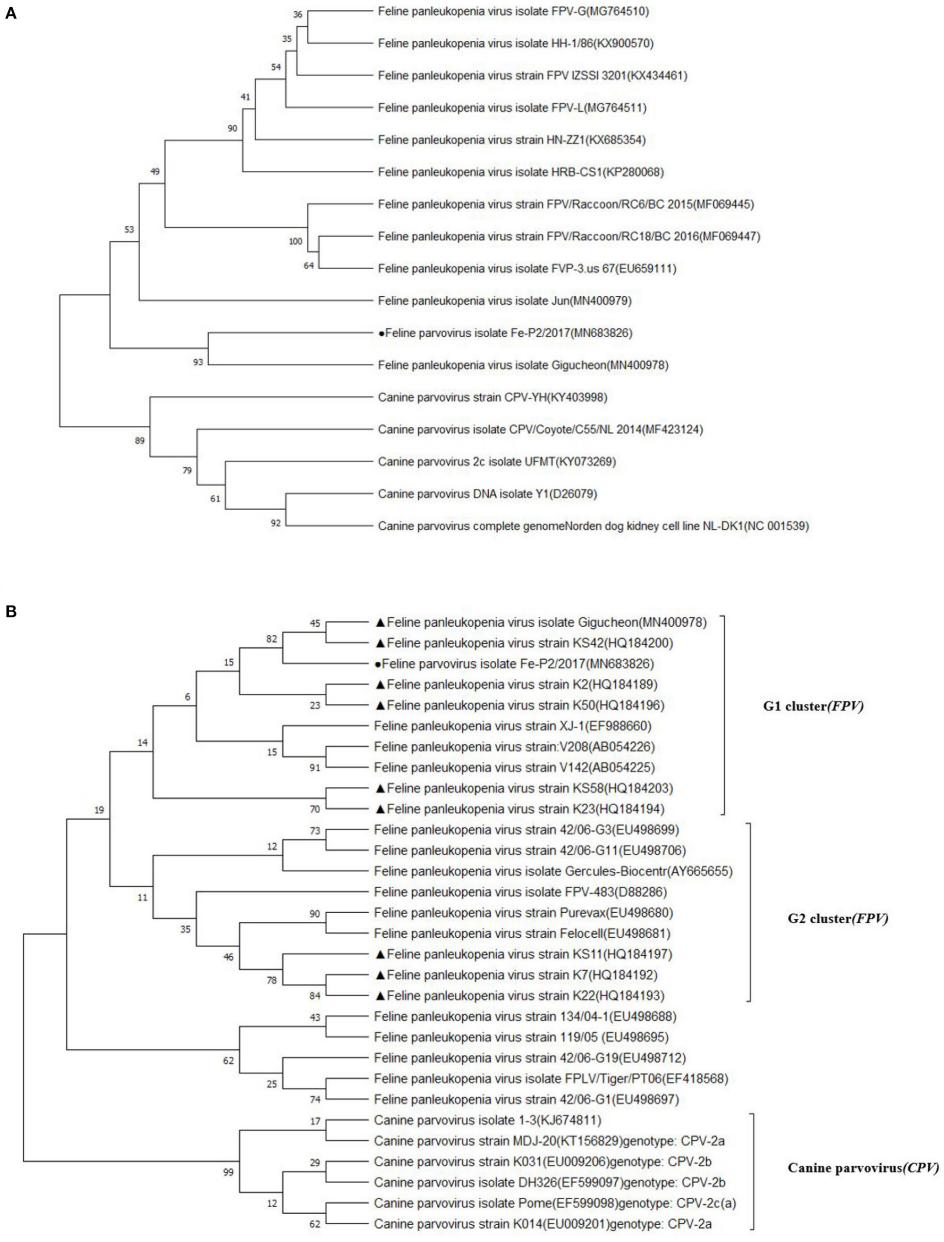
**(A)** Phylogenetic tree based on partial genomic sequences; black solid circle indicates the FPV Fe-P2 virus. The tree produced by the maximum likelihood method using MEGA 7.0 software shows the phylogenetic relationship between the 5 canine parvovirus strains and 12 feline parvoviruses. **(B)** Phylogenetic tree based on VP2 nucleotide sequences; black solid circle indicates the Fe-P2 virus, and black solid triangles indicate viruses isolated in Korea. The tree produced by the maximum likelihood method using MEGA 7.0 software shows the phylogenetic relationship between the 6 canine parvovirus strains and 24 feline parvoviruses.

The partial genomic sequence of 4,643 bases of FPV Fe–P2 strain was obtained in this study. Two main open reading frames (ORFs) encoding VP1 and VP2 were deposited as complete coding sequences. As a determinant of the host range of parvoviruses, it appears to be the minority amino acids of the capsid protein that determine the ability of the virus to replicate in different hosts (3). The host-specific amino acid position in VP2; 80, 93, 103, 297, 300, 305, 323, 564, and 568 (6) were compared with other FPVs and CPVs. All the amino acids were well-conserved among FPVs including the Fe–P2 strain, although these differed from CPV-2a, 2b, and 2c strains (Table 3). The partial genomic sequence-based phylogenetic tree was drawn with other strains of *Carnivore protoparvovirus 1* (Figure 2A). Based on the phylogenetic relationship, it is suggested that the FPV Fe-P2 strain was not related to the recently reported FPV from wild raccoon dogs (GenBank Accession No. MF069445 and MF069447) in Canada (16). Phylogenetic analysis based on the VP2 nucleotide sequence showed that the FPV Fe–P2 strain belonged to the G1 cluster (Figure 2B). The FPV Fe–P2 in this study was closely related to a feline-specific FPV strain (GenBank Accession No. MN400978), which was reported previously in Korea (Figure 2B), showing 99.16% sequence identity.

**Table 3:** Amino acids positions for host-specificity between dogs and cats.

**Virus strain**	**Accession number**	**Amino acid VP2**								
		**80**	**93**	**103**	**297**	**300**	**305**	**323**	**564**	**568**
Feline parvovirus Fe-P2^*^	MN683826^*^	K	K	V	S	A	D	D	N	S
Feline parvovirus (FPV)	HQ184189	K	K	V	S	A	D	D	N	S
	AB054226	K	K	V	S	A	D	D	N	S
	KP081409	K	K	V	S	A	D	D	N	S
	KJ415112	K	K	V	S	A	D	D	N	S
Canine parvovirus (CPV)-2a	EU009201	R	N	A	A	G	Y	N	S	R
	KT156829	R	N	A	A	G	Y	N	S	R
Canine parvovirus (CPV)-2b	FJ977077	R	N	A	A	G	Y	N	S	R
	EF599097	R	N	A	A	G	Y	N	S	R
	EU009206	R	N	A	A	G	Y	N	S	R
Canine parvovirus (CPV)-2c(a)	EF599098	R	N	A	A	D	Y	N	S	R

* *The partial genomic sequence of 4,643 bases from Feline Parvovirus Fe-P2 (MN683826)*.

### Serum Neutralizing Antibodies Against FPV Fe-P2 in Leopard Cat and Asian Badger

In the result of the SN test against FPV Fe-P2 with a total of 109 sera from wild mammals (section Serum Neutralization Test), the neutralizing antibodies were evident in the sera of Asian badgers and wild leopard cats, showing SN titer, 80–1,280 (Table 4). Five out of eight leopard cats and all the Asian badgers (two samples) were seropositive with the SN test. The other wild mammals, including 96 Raccoon dogs, one water deer, one Eurasian otter, and one Korean hare did not have SN titers more than 20 against FPV Fe–P2. The SN titers in Asian badger were 80 and 320, while that in leopard cats were between 80 and 1,280.

**Table 4:** Serum neutralization test results.

**Animal species**	**Year**	SN-positive/total sera^*^	**SN titer of the positive**
**Raccoon dog** (*Nyctereutes procyonoides koreensis*)	2016–2018	0/96	<20
**Leopard cat** (*Prionailurus bengalensis*)	2016–2018	5/8	80, 1,280, 1,280, 1,280, 1,280
**Asian badger** (*Meles leucurus*)	2016–2017	2/2	80, 320
**Korean hare** (*Lepus coreanus*)	2017	0/1	<20
**Water deer** (*Hydropotes inermis*)	2018	0/1	<20
**European otter** (*Lutra lutra*)	2018	0/1	<20
	Total (sera)	7/109	

* *Five out of eight leopard cats and two Asian badgers were seropositive with the SN test*.

## Discussion

FPV-infected cats older than 6 weeks develop symptoms from subclinical level to sudden death within 12 h (5). FPV-infectious disease is characterized by severe panleukopenia and enteritis, which is also associated with high mortality and morbidity (3, 5, 17). Multiple, epizootic outbreaks of FPV infection in most unvaccinated cats were reported in Australia between 2014 and 2018 (2). In Korea, FPV infection was found in 2% of cats in Seoul (11), and FPVs belonging to the G1 and G2 clusters were circulating (12). In this study, an FPV isolate Fe–P2 from 2017 was included in the G1 cluster, and amino acids of the VP2 protein indicate that the isolate would have the same host specificity as other FPVs. Observing no close relationship with recent novel strains of FPV from wild raccoon dogs in Canada (16), the FPV isolate Fe–P2 in this study might be one of the strains circulating in Korea. In addition, the amino acids positions for host-specificity of Fe–P2 strain were the same as other feline parvoviruses rather than canine parvoviruses. There were no apparent genetic differences between Fe-P2 strain and other FPVs. Although there should be additional screening on the prevailing FPV genotypes in Korea, we used this isolate to screen the serological evidence of FPV infection in wild animals.

As we can collect sera of six wild animals from the Chungnam Wild Animal Rescue Center, we tried to screen SN antibodies using the recent FPV isolate, Fe–P2 in this study. As expected, no SN antibodies were observed in Korean hare and water deer, which could be regarded as negative controls for the SN test in this study. Notably, two wild mammals, leopard cat and Asian badger were seropositive in the SN test using the FPV isolate Fe–P2 with 109 sera of wild mammals from Korea. The SN titers were notable due to relatively high titers, 80 and 1,280 in leopard cats and 80 and 320 in Asian badgers.

Parvoviruses have shown consistently evolution over many years at a faster rate than other DNA viruses. Also, a variety of wild animals have increasingly detected as parvoviruses hosts (8). FPV has been known to infect other species like raccoon dogs, foxes, minks, African wild cats, cheetahs, and honey badgers (5, 6). Furthermore, FPV was still frequently found in wild animals like otters, minks, and martens (18, 19). The FPV-positive rate in Canadian otters was almost 30%. Hence, it was suggested that otters might be a principal maintenance host for FPV enabling viral persistence and serving as a source for other susceptible species (18). In this study, the serum from otter had SN titer <20 in the SN test against FPV Fe-P2 strain. However, since only one otter sample was tested, there may be a possibility for FPV infection in the species, additional screening would provide more detailed information.

In this study, five out of eight leopard cats were seropositive against the FPV Fe–P2 strain, which may indicate they were infected by FPV previously. This is not surprising as FPV had already been detected in leopard cats in Taiwan and Vietnam (20, 21). However, seropositive Asian badgers (two samples) in the SN test against FPV Fe–P2 strain, were identified in this study for the first time. While FPV was detected in honey badger (*Mellivora capensis*) in South Africa (6), in this study we report the serological evidence of FPV infection in Asian badgers (*Meles leucurus*), which belong to a different subfamily that had never been reported before. Thus, this study might provide partial evidence for the possible circulation of FPV or its related viruses among wild leopard cat and Asian badger in Korea.

As a variant of *Carnivore protoparvovirus 1*, FPV had wide range of host animals. Evidence for FPV infection in a diverse range of wild mammals indicated that FPV has been circulating not only in cats but also in other mammals, with sporadic interspecies transmission. In this study, we could suggest Asian badgers and wild leopard cats as potential hosts for FPV infection in Korea. However, conventional parvoviruses may have observed cross-reactivity through high virus-neutralizing (VN) antibodies titers between FPV and CPV (22), thus we cannot exclude the possible infection of CPVs in those animals. There should be additional study to confirm this through direct detection of FPVs in the related animal samples. Therefore, it would be more feasible that there was serological evidence of FPV or its related viruses in Asian badgers and wild leopard cats in Korea.

Thus, FPV and its related viruses may circulate in the wild life hosts. The leopard cats and Asian badgers in this study were rescued animals from human populated regions, which means that they were near human habitat. Therefore, a vaccination or a control policy against FPV and its related viruses should be considered not only for household cats, but also for wild animals.

## Data Availability Statement

The datasets presented in this study can be found in online repositories. The names of the repository/repositories and accession number(s) can be found in the article/supplementary material.

## Ethics Statement

Ethical review and approval was not required for the animal study because we used the archived sera which were collected for veterinary treatment from the rescued wild animals by Chungnam Wild Animal Rescue Center in Korea.

## Author Contributions

HK and JJ: conceptualization. YK and S-WY: methodology. JJ: resources. DJ: data curation. YK and HK: writing—original draft preparation. BL, S-WY, and DJ: writing—review and editing. BL and S-WY: supervision. HK and DJ: funding acquisition. All authors contributed to the article and approved the submitted version.

## Conflict of Interest

The authors declare that the research was conducted in the absence of any commercial or financial relationships that could be construed as a potential conflict of interest.
